# Mother and daughter with adolescent-onset severe frontal lobe dysfunction and epilepsy

**DOI:** 10.1590/S1980-5764-2016DN1003011

**Published:** 2016

**Authors:** Giordani Rodrigues dos Passos, Alonso Cuadrado Fernández, Adriana Machado Vasques, William Alves Martins, André Palmini

**Affiliations:** 1 Neurology Service, São Lucas Hospital, Pontifical Catholic University of Rio Grande do Sul, Porto Alegre RS, Brazil.; 2 Institute of Geriatrics and Gerontology, Pontifical Catholic University of Rio Grande do Sul, RS, Porto Alegre, Brazil.; 3 Porto Alegre Epilepsy Surgery Program, Neurology Service, São Lucas Hospital, Pontifical Catholic University of Rio Grande do Sul, Porto Alegre, RS, Brazil.

**Keywords:** frontal lobe, genetics, behavioral, neuropsychiatry, epilepsies, partial, heredity

## Abstract

Familial cases of early-onset prominent frontal lobe dysfunction associated with epilepsy have not been reported to date. We report a mother and her only daughter with incapacitating behavioral manifestations of frontal lobe dysfunction and epilepsy of variable severity. The possibility of a hitherto undescribed genetic condition is discussed.

## RESUMO

Casos familiares de disfunção proeminente do lobo frontal associada a epilepsia com início precoce ainda não foram relatados. Nós descrevemos uma mãe e sua filha única com manifestações comportamentais incapacitantes de disfunção do lobo frontal e epilepsia de severidade variável. A possibilidade de uma condição genética ainda não descrita é discutida.

## INTRODUCTION

Frontal lobe dysfunction is nonspecific and may be the presenting feature of many different brain insults. Some are obviously acquired and indeed classical texts in Neurology based the description of the 'frontal lobe syndrome' on patients with extensive traumatic, neoplastic, vascular or degenerative destruction of the frontal lobes.^1^ However, a number of genetically-determined conditions have been increasingly reported, mostly associated with degenerative or metabolic diseases, often presenting in late adulthood and having unequivocal progression.^2^ Furthermore, when disorders predominantly involving the frontal lobes present early in life, they usually have additional neurological features, such as white matter progressive abnormalities and motor dysfunction.^2^ Thus, earlier-onset prominent frontal lobe syndrome unaccompanied by overt motor or imaging abnormalities is apparently rare and even more so with a familial presentation. 

Genetic factors are being increasingly recognized as underlying many types of epilepsy. Some constitute severe epileptic encephalopathies and others focal forms of epilepsy, previously considered cryptogenic.^3^ Some of the latter may involve the frontal lobes without overt imaging abnormalities, but are not specifically associated with severe frontal lobe-related behavioral and emotional dysregulation. For instance, autosomal dominant nocturnal frontal lobe epilepsy (ADNFLE), a type of frontal lobe epilepsy with proven genetic origin, is in fact a relatively benign entity, whose seizures can be easily controlled using antiepileptic medication and usually associated with normal cognition and behavior.^4^ ^,^ ^5^ Other types of frontal-predominant epilepsies of genetic origin include gross malformations of cortical development, such as bilateral frontal polymicrogyria, which can be inherited and occur in families.^6^ Hence, genetic forms of frontal lobe epilepsy without gross imaging abnormalities and with incapacitating behavioral manifestations consistent with frontal lobe dysfunction are apparently rare and have not been reported to date. 

Here we report a mother and daughter with a largely similar, challenging incapacitating non-degenerative frontal lobe syndrome associated with nonlesional, pharmacoresistant frontal lobe epilepsy. Their condition is apparently unique in that severe frontal lobe dysfunction is associated with epilepsy, normal imaging and a likely autosomal dominant genetic substrate. Furthermore, more than 20 years´ follow-up of both patients have provided us the rare opportunity of a longitudinal assessment. Because the patients were deemed legally incapable, written informed consent for case reporting was obtained from their legal guardian. 

## CASE REPORTS

The mother. This is a 49-year-old Caucasian woman with no history of parental consanguinity or family history of neuropsychiatric disorders. Pregnancy, delivery and early development were uneventful and there was no history of febrile seizures. When she entered school, agitation, learning difficulties and relationship problems became apparent. At age 10 she began having weekly diurnal seizures with an aura of "feeling as if I am about to lose control", followed by hemiclonic movements in alternating sides and secondary generalization. Seizures were difficult to control with medication, but the most striking aspect of her adolescence was a marked behavioral worsening. Despite a good sociocultural background, she started to frequently engage in promiscuous relationships, mostly with men she met in the streets. This later deteriorated into bouts of verbal aggression, threats to relatives and neighbors with a knife and stealing objects, leading to problems with the law. At age 20 years, she fell pregnant and abandoned her parents' house to live with her partner in a setting involving extreme poverty and the need for begging. She soon demonstrated negligent and aggressive behavior towards her daughter, resulting in a court decision deeming her incapable of raising a child, which led to sterilization. 

**Figure 1:**
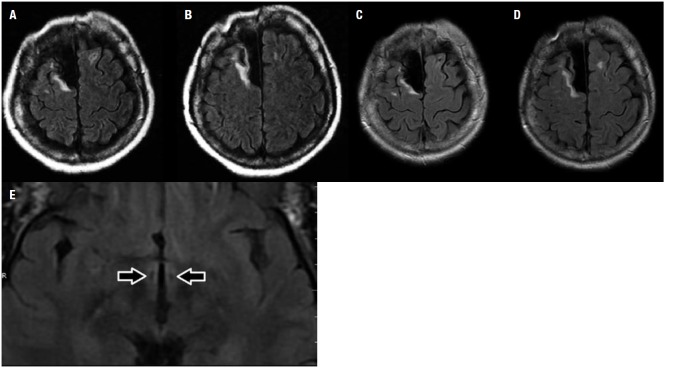
Mother's FLAIR MRI of the brain at 41 (A and B) and 49 years (C, D, and E) of age. Note resection of the right superior frontal gyrus with surrounding gliotic border, as well as small, non-specific hyperintense and hypointense subcortical lesions in the left frontal lobe (A to D). Notably, there is no major atrophy and images are very similar, following the 8-year interval. Note the surgical lesion at the posteromedial hypothalamus bilaterally (E, arrows).

In parallel with this severe frontal lobe dysfunction and psychopathic social functioning, seizure control progressively worsened. She was initially seen at our Center at age 26 and referred for presurgical assessment at age 29, following unequivocal documentation of seizure refractoriness. Scalp electroencephalography (EEG) showed intense epileptiform discharges over the right frontotemporal regions, with much less intense discharges in the homologous area of the left hemisphere. Presurgical neuroimaging with magnetic resonance imaging (MRI) was negative, leading to invasive investigation with intracranial electrodes. Invasive EEG recordings showed bilateral frontal epileptiform activity, much more pronounced in the right side, where seizures were recorded. She had resection of the right superior frontal gyrus and anterior cingulate cortex (Figure 1), guided by intraoperative electrocorticography. Histopathological examination of the resected brain sample showed only astroglial proliferation and relative reduction in neuronal population, with no evidence of focal cortical dysplasia or inflammation. 

Following the surgery, there was marked improvement in seizures, which were completely controlled over the years. In contrast, behavioral problems continued to worsen and also proved refractory to all medication attempts. The most dramatic episode was when she threw boiling water into her 8-year-old daughter's face, which led to loss of custody of the child. At this time, and on two other occasions, she was admitted to psychiatric wards. Due to exhaustion of all medication and behavioral therapeutic strategies, she underwent simultaneous bilateral posteromedial hypothalamotomy (Figure 1) at age 37, with the aim of decreasing violent behavior. Despite improvement in aggressiveness, she remained incapable of social functioning, being transferred to a long-term nursing care facility one year later. 

Currently, she is on carbamazepine 1200 mg/day and has been seizure-free for one year. She is also on quetiapine (50 mg/day and increasing), but serious non-adherence to social norms and persistent aggressiveness remain as major concerns. 

Neuropsychological tests performed at age 28 and again at age 49 were compared (Table 1). On the first assessment (before epilepsy surgery), intelligence was rated as average to low average; there were significant deficits in attention and speed of processing, memory, learning, and executive functions; while no visuospatial or language dysfunction was noted. Twenty-one years later, there was marked worsening of attention and speed of processing, slight worsening of memory, and appearance of visuospatial deficit, while intelligence, learning, and executive deficits remained fairly stable.

**Table 1:** Neuropsychological assessment of the mother (at different ages) and the daughter.

Cognitive domains / specific tests*		Mother - at age 28^§¶^	Mother - at age 49^¶^	Daughter - at age 28^§^
Memory - Wechsler Memory Scale - Revised (SD)	Verbal Memory I	-0.8	-1.6	-1.8
	Verbal Memory II	-1.3	-1.8	-0.9
	Visual Memory I	0.2	-3.6	-0.7
	Visual Memory II	-3.2	-3.8	-1.1
Verbal learning - Rey Auditory Verbal Learning Test (SD)	Verbal Learning I	-1.0	-1.7	-1.7
	Verbal Learning II	-2.1	-1.1	-1.3
Intelligence - Wechsler Adult Intelligence Scale (raw scores and corresponding categories)	Arithmetic	7 / low average	7 / low average	6 / low average
	Digit Span	11 / average	11 / average	7 / low average
	Similarities	11 / average	9 / average	10 / average
	Comprehension	10 / average	6 / low average	4 / low
	Block Design	8 / low average	7 / low average	8 / low average
	Picture Completion	8 / low average	10 / average	11 / average
	Picture Arrangement	8 / low average	9 / average	9 / average
	Digit Symbol	7 / low average	7 / low average	7 / low average
Attention and processing speed - Trail Making Test (SD)	Test A	-3.9	-16.5	-2.7
	Test B	-3.2	-12.3	-7.9
Executive function	Wisconsin Card Sorting Test (raw score)	3 out of 6	3 out of 6	0 out of 6
Visuospatial ability	Rey-Osterrieth Complex Figure Test (SD)	0.2	-8.0	-2.6
	Hooper Visual Organization Test (category)	superior	very superior	N/A
Language	Boston Naming Test (SD)	1.1	0.5	0.9

*Scores highlighted with gray background indicate deficits on the neuropsychological tests. ^§^Note the similar pattern of neuropsychological dysfunction for both mother and daughter at age 28 years. ^¶^Note the progression of the mother's cognitive deficits from age 28 to age 49. SD: standard deviation; N/A: not available.

**Figure 2:**
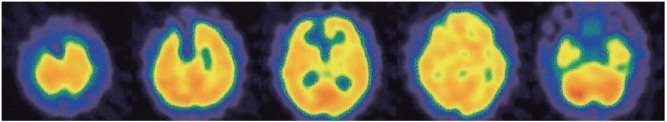
Mother's recently obtained 99mTc-ECD brain perfusion SPECT axial images. Except for the absence of perfusion over the surgically resected area in the right frontal lobe, brain perfusion is normal.

Besides neuropsychiatric dysfunction and epilepsy, there were no other significant neurological abnormalities in the motor, sensory, language or visual domains. She also had no evidence of systemic comorbidity. Throughout follow-up, a number of paraclinical tests were done, including cell blood count, renal, liver and thyroid function tests, serum glucose, lactate, arterial blood gas, and serology for viral hepatitis, syphilis and HIV, all within normal limits. Following the surgical procedures for epilepsy and violent behavior, MRI of the brain was performed at ages 41 and 49 years; besides the postsurgical findings in the right frontal lobe and in the posteromedial hypothalami, there were small, non-specific subcortical lesions in the left frontal lobe (Figure 1). Notably, there was no progressive atrophy or other evidence of neurodegeneration on MRI. Brain perfusion single-photon emission computerized tomography (SPECT) was also performed, at age 49 years, with no apparent areas of hypoperfusion (Figure 2), except for the area corresponding to the previous surgical resection.

The daughter. This 28-year-old mestizo woman is the only daughter of the patient reported above with a non-consanguineous spouse. During pregnancy, she was exposed to anticonvulsant, sedative, antipsychotic and recreational drugs, as well as maternal seizures. There were no pre- or perinatal events, congenital malformations or neurodevelopmental delay. There was no history of febrile seizures. During childhood, she suffered all sorts of violence committed by her parents, from whom she was legally separated at age 8 years. At this age, she also started exhibiting learning difficulties and aggressive behavior. 

Between ages 8 and 11 years she had infrequent diurnal, poorly described seizures. Complete seizure control was achieved after initiation of phenytoin. Throughout adolescence, she gradually developed severe frontal lobe dysfunction, including disinhibition (used to undress in public), puerile behavior (would rather play with children than interact with other teenagers), psychomotor agitation (used to climb furniture and run and jump relentlessly around her house), impaired judgement (engaged in promiscuous relationships with married, homeless, drug-addicted and delinquent men), lack of impulse control, aggressiveness and total lack of hygiene and self-care. 

Psychiatric treatment started at age 12. Hormonal therapy with injectable progestogen was required in order to control sexual impulses. She later developed prominent anxiety symptoms, including panic attacks and compulsive behaviors, such as hair pulling (eventually leading to significant alopecia) and pushing the flush dozens of times after using the toilet.

**Figure 3:**
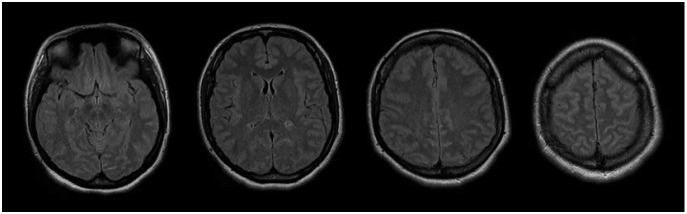
Daughter's FLAIR MRI of the brain obtained at age 28 years. Images at different levels show no evidence of frontal lobe lesions, signal abnormalities or atrophy.

As in her mother's case, there were no other neurological symptoms or signs. Apart from obesity and dyslipidemia, there were no other systemic diseases. Multiple scalp EEGs showed relatively synchronous epileptiform discharges in the anterior regions of both hemispheres, suggestive of frontal lobe epilepsy, some with clear left frontal predominance. Brain MRI was unremarkable at age 20 years and again at age 28 (Figure 3), with no significant brain atrophy having developed during that interval. 

After many failed attempts with multiple psychotropic drugs, satisfactory control of the behavioral symptoms and some improvement in cognitive function were ultimately achieved with the combination of clozapine 100 mg/day, aripiprazole 20 mg/day, paroxetine 20 mg/day, and diazepam 15 mg/day. Despite having been deemed legally incapable at age 23, her ability to live at home is preserved, under the supervision of her grandparents, and she does engage in some self-care and house-keeping activities. In addition, she has been seizure-free on phenytoin 400 mg/day. 

Neuropsychological assessment at current age (Table 1), with the same tests used for her mother's cognitive evaluation, showed average to low average intelligence, as well as deficits in attention and speed of processing, memory, learning, visuospatial abilities and particularly executive functions, with no language dysfunction.

## DISCUSSION

We report a family in which a mother and her only daughter presented with progressive and incapacitating frontal lobe dysfunction and epilepsy of variable severity. Interestingly, age of onset of the neuropsychiatric symptoms (6-8 years) and of seizures (8-10 years) were similar in the two patients, as was the course of the behavioral and cognitive worsening, which was relatively rapid throughout adolescence and seemed to plateau during the third decade, with progression at a slower rate. Moreover, neuropsychological profile on formal testing, including severity of the deficits, was quite similar for both mother (before surgery) and daughter, at age 28 years. Finally, refractoriness to several psychotropic drugs was another common point. In contrast, gestation and upbringing were very different. Whereas the mother was the product of an uneventful pregnancy and raised in a caring environment, the daughter had significant drug exposure during gestation, meager prenatal care and suffered severe abuse and neglect during childhood. Thus, this pattern of very similar clinical evolution, despite markedly different environmental upbringing, does suggest a genetic condition.

Executive functions include attentional and inhibitory control, working memory, cognitive flexibility, reasoning, problem solving, and planning.^7^ Even though the prefrontal cortex seems to be the main anatomical substrate of these higher-order cognitive processes, the historical linkage of executive functions and the frontal lobes has been reformulated in order to recognize that a one-to-one relationship between structure and function is not possible and other anatomical sites also play a role.^7^ ^,^ ^8^

Although psychiatric comorbidities of epilepsy are currently well studied,^9^ ^,^ ^10^ the specific pattern of behavioral and cognitive dysfunction reported here could not be explained solely by any given psychiatric disorder, such as personality disorder, bipolar disorder or schizophrenia. Furthermore, frontal lobe epilepsy, even when refractory to medication, does not usually manifest with such severe degree of neuropsychiatric frontal lobe dysfunction, except when seizures accompany frontal lobe destruction, such as those following severe head trauma, tumor resection or massive parenchymal hemorrhage.^11^ ^,^ ^12^ It is known that people with frontal lobe epilepsy may have behavioral abnormalities, including hyperactivity, conscientiousness, obsession, and addiction,^11^ but not to the degree seen in these two patients, which resemble classical, severe frontal lobe syndromes with marked disinhibition and executive dysfunction.^1^ ^,^ ^7^ ^,^ ^13^

Frontal lobe dysfunction can result from several conditions, many of which may have a genetic cause.^2^ Frontotemporal dementia (FTD) and Alzheimer's disease may have familial presentations, manifesting at an earlier age in comparison to sporadic cases.^14^ In a series of FTD spectrum diseases, Le Ber et al. reported onset of symptoms in a patient with MAPT mutation at age 17 years, which is, however, exceedingly rare.^15^ Moreover, these degenerative familial disorders usually do not include epilepsy as a prominent feature. Another group of conditions that should be considered in the differential diagnosis of our patients include genetically-determined neurodegenerative disorders, such as Huntington's disease and dentatorubropallidolusyian atrophy, or metabolic diseases, such as Niemann-Pick disease type C and leukodystrophies.^2^ These may have childhood onset, be associated with epilepsy and progress with severe behavioral and cognitive abnormalities. However, these entities are usually associated with a number of other features, especially motor or visual abnormalities, brain MRI findings and systemic signs and symptoms, which were absent in the cases reported here.^2^ ^,^ ^14^

Genetic variation has been increasingly recognized as a major etiology of epilepsies.^3^ Several single genes whose mutation is clearly associated with epilepsy have already been described and there has been a spate of discoveries, particularly in relation to epileptic encephalopathies.^16^ ^-^ ^18^ The latter are entities in which the extremely high frequency and severity of the epileptic seizures and interictal epileptiform discharges lead to progressive cognitive and behavioral deterioration. Although these encephalopathies almost by definition begin very early in life, the pattern of evolution differs distinctly to that of our patients in that the epilepsy is much more severe and development is always delayed from very early in life. In many other instances of probably genetic-related epilepsies, a single genetic mutation cannot be identified; instead, multiple genes and modulation of genetic expression by environmental factors likely play a role.^3^

These aspects notwithstanding, two genetically-determined epilepsy syndromes were considered in the differential diagnosis of our cases. ADNFLE is caused by mutations in the genes CHRNA2, CHRNA4 or CHRNB2, inherited in an autosomal dominant manner, with 70% penetrance, and presents during the first two decades of life usually with nocturnal focal seizures.^19^ Familial partial epilepsy with variable foci is caused by autosomal-dominant mutations in the gene DEPDC5 and usually manifests as familial cases of epilepsy in which each individual may have a different single focus.^16^ Both conditions could explain epilepsy in our patients, but would not be expected to account for the early, prominent and incapacitating frontal lobe dysfunction. Moreover, both the mother and the daughter had predominantly diurnal seizures, making the diagnosis of ADNFLE less likely. 

To our knowledge, the familial occurrence of severe frontal lobe dysfunction and epilepsy, with onset in the first decade of life, is extremely atypical and warrants further investigation. We are well aware that further histopathological and genetic workup would be of paramount importance to establish a diagnosis for the cases reported here. Unfortunately, with 20 years having passed since the mother underwent epilepsy surgery, we were not able to recover the pathological samples, thus immunohistochemistry analysis and other molecular techniques currently available could not be performed. We do not know whether mild foci of type I focal cortical dysplasia or other relevant pathological findings would be disclosed if these analysis were performed. In addition, a comprehensive genetic panel for epilepsy would be of value, but was not possible to perform due to practical issues. Currently, exome sequencing is being planned and may potentially lead to the identification of a novel mutation accounting for the development of adolescent-onset severe frontal lobe dysfunction associated with epilepsy. 
